# Supplementary material for: Learning curve and surgical outcome of robotic assisted colorectal surgery with ERAS program
Source: Sci Rep. 2022 Nov 29;12:20566. doi: 10.1038/s41598-022-24665-w (PMC9709162; doi:10.1038/s41598-022-24665-w)
Supplement: Supplementary file 2 — Supplementary Information 2. [file 41598_2022_24665_MOESM2_ESM.zip › RAL Raw data-Ñ[▒K/072/HS_ñw╝╨░O▒Kñσ.pdf]

正式摘要(電子簽章：000000214282946)	□【讀取】憑證	
W92 / W92 □英文標題(注意:無英文姓名-請補病人基本檔!)		

臺中榮民總醫院
出院病歷摘要

轉入號： 轉出號：

[redacted]

(8)流水編號	
(9)入院日期	110/04/07 (2021) 直腸外科 W92 - 37
(10)轉科日期	
(11)出院日期	110/04/12 (2021) 住院天數計 5 日

診斷

- 入院:1. Suspicious mass lesion over para-rectum area
- 出院:1. Suspicious mass lesion over para-rectum area, s/p robotic low anterior resection on 2021/04/08

主訴

- for ROBOTIC TME FOR PARA-RECTAL TUMOR

病史

- This 46 year old male had past history with
  1. Peritonitis with ruptured appendix s/p laparoscopic appendectomy on 2021/01/15
  2. Hypertension under medication control
  3. Diabetes mellitus under medication control
  He had peritonitis with ruptured appendix s/p laparoscopic appendectomy on 2021/01/15, and at that time, rectal mass was found accidentally in abdominal CT. He was referred to our CRS OPD for further survey. He had no fever, no anorexia, no abdomial pain, no body weigth loss. This time, he was admitted for surgical intervention.
  Bokey hold >7 days

體檢發現

-
  - General appearance :
    - easy looking , conscious clear , without cardiopulmonary distress
    - Vital signs自動帶入date 體溫(BT) 脈搏(P) 呼吸(R) 血壓(BP) 身高(BH) 體重(BW) BMI
      20210407 37℃ 102次/分 18次/分 146/77mmHg 178.2cm 123.9kg 39.0
    - Vital signs: B.P. - mmHg , P.R. 5 /min , R.R. 5 /min
  - HEENT :
    - Eyes: no anemic conjunctiva, no icteric sclera, normal EOM
    - ENT: no otorrhea, no rhinorrhea, no erythematous mucosa, no injected tonsil, no deformity
  - Neck: supple, freely movable, trachea midline,no deformity, no engorged jugular vein , no palpable thyroid or lymph node enlargement
  - Chest:
    - Inspection: Grossly normal, symmetric and fully expansion, no accessory muscle used, no operation scar, no spider angiomata, no gynecomastia
    - Palpation: no palpable mass or nodule, no subcutaneous emphysema, no tenderness
    - Ausculation: clear breathing sound
  - Heart:
    - Percussion: normal heart size
    - Ausculation: regular heart beat, normal S1, S2, no gallop, no murmur
  - Abdomen:
    - Inspection: in
      - flat contour, symetrically, no scar, no stria, no dilated veins, no rashes
    - Ausculation: normoactive bowel sound, no abdominal bruits
    - palpation: soft, no muscle guarding or rigidity, no local tenderness, no rebounding pain, no palpable mass, no palpable pulsation

- o Percussion: no shifting dulllness, no hepatomegaly, no splenomagaly
-

Peripheral pulsation :	CA	BA	RA	FA	PA	PTA	DPA
Right side :	++	++	++	++	++	++	++/+++
Left side :	++	++	++	++	++	++	++/+++

- Genito-anus: no tenderness or palpable mass lesion or rectal shelf, normal external genitalia, normal anal tone, brown stool

CRS CONDITION

-
    - o Lung

手術日期及方法

- 手術日期及方法20210408 1. Robotic (XI) assisted low anterior resection
  2. Intraoperative colonoscopy : (-).
  3. High ligation (-) : IMA was not approached.
  4. Take down from the splenic flexure : (-)
  5. Robotic EndoGIA (45-4.3) X2 + EEA (33mm) were applied to complete the end-to-end anastomosis
  6. Proximal cut end: 5 cm, distal cut end: 2 cm
  7. A 12 Fr. penrose drainage tube was inserted from RLQ of abdomen into the pelvic
  8. ADHESION BARRIER FILM was placed below the centrol extended wound
- 手術結果及發現 :
    - 1. A 5 x 4 cm tumor at para-rectum area. It may be originated from the rectum ? 2. Severely adhesion was noted below the umbilicus, which was compatible with the hx of s/p laparoscopic operation for ruptured appendicitis 3. No other gross tumor nor polyps was found 4. There was no obvious visible liver metastasis, no visible peritoneal seeding 5. No obvious ascites was noted

住院治療經過

- After admission, pre-op survey was done and there was no contraindication for opeartion. Robotic low anterior resection was performed smoothly on 04/08 and he tolerated the procedure. After OP, he recovered well and no post-op complication was noted. Oral intake was smooth since 04/09. Under the stable condition, he was discharged and would be followed-up at OPD.

合併症

- nil

一般檢查

- 累積報告 :

DATE	TIME	ALB	TP	BIL.T	BIL.D	ALKP	AST	ALT	LDH	CHOL	TG	HDL-C	LDL	UA	NH3	AMY
1100408	1644	3.8		0.97	0.37		76	58								

DATE	TIME	WBC	RBC	HGB	HCT	MCV	PLT	BLST	NEUT	BAND	SEG	LYM	MON	EOS	BAS
1100408	1644	12710		14.3	40.9		178		91.3			5.2	1.6	1.7	0.2
1100412	0650	8950	4.93	13.9	41.4	84.0	188		74.2			18.5	6.0	0.9	0.4

DATE	TIME	NA	K	CL	CA	BUN	CREAT	Mg	P	CRP	ESR	PCT	LIPASE	LACTATE
1100408	1644	137	3.6		8.8	15	0.88							
1100412	0653	139	3.8			13	0.59							

DATE	TIME	GLUCOSE	PAB	TRF	ZN	CU	KETONE	CK	CKMB	PT-P	PT-C	APTT-P	APTT-C	TRO-T
1100412	0649									11.2	10.6	25.9		

DATE	TIME	SP-GR	PH	PROT	GLUC	KETON	BILI	UROB	OB	NITR	LEU	RBC	WBC	EPCEL
1100408	1741	1.028	5.5	Trace	>1000.0	Trace	Negative	0.2	Trace	Negative	Negative	2-5	2-5	0-1

特殊檢查

病理報告

- Pending

其它

出院時情況

- 3.改門診治療

出院指示

藥名	劑量	劑量單位	用法	頻次	數量	數量單位	現況	天數	單位
(口)Takepron OD tab 30mg	1	TAB	PO	QDAC	7	TAB	TH	7	天
Ultracet tab	1	TAB	PO	Q6H	28	TAB	TH	7	天
Cap Trand 250mg	1	CAP	PO	QID	12	CAP	TH	3	天

出院計劃

主治醫師 2062F 蔣鋒帆 / 住院醫師 2334J 陳勇先

正式摘要**(電子簽章：000000214282946)**

W92 / W92

doctorRank:B
